# CD28 Family and Chronic Rejection: “To Belatacept...and Beyond!”

**DOI:** 10.1155/2012/203780

**Published:** 2012-06-07

**Authors:** Marcos V. Silva, Juliana R. Machado, Laura P. Rocha, Lúcio R. Castellano, Marlene A. Reis, Rosana R. M. Corrêa

**Affiliations:** ^1^Immunology Laboratory, Department of Biological Sciences, Federal University of Triângulo Mineiro, Uberaba, MG, Brazil; ^2^Pathology Laboratory, Department of Biological Sciences, Federal University of Triângulo Mineiro, 30 Frei Paulino Street, Abadia, 38025-180 Uberaba, MG, Brazil; ^3^Technical Health School of UFPB, Federal University of Paraíba, João Pessoa, PB, Brazil

## Abstract

Kidneys are one of the most frequently transplanted human organs. Immunosuppressive agents may prevent or reverse most acute rejection episodes; however, the graft may still succumb to chronic rejection. The immunological response involved in the chronic rejection process depends on both innate and adaptive immune response. T lymphocytes have a pivotal role in chronic rejection in adaptive immune response. Meanwhile, we aim to present a general overview on the state-of-the-art knowledge of the strategies used for manipulating the lymphocyte activation mechanisms involved in allografts, with emphasis on T-lymphocyte costimulatory and coinhibitory molecules of the B7-CD28 superfamily. A deeper understanding of the structure and function of these molecules improves both the knowledge of the immune system itself and their potential action as rejection inducers or tolerance promoters. In this context, the central role played by CD28 family, especially the relationship between CD28 and CTLA-4, becomes an interesting target for the development of immune-based therapies aiming to increase the survival rate of allografts and to decrease autoimmune phenomena. Good results obtained by the recent development of abatacept and belatacept with potential clinical use aroused better expectations concerning the outcome of transplanted patients.

## 1. Introduction

Kidneys are one of the most frequently transplanted human organs, with approximately 10,000 kidney transplants being performed annually in the United States [1]. Regarding absolute numbers of kidney transplantations, Brazil ranks second among all countries, after the United States and ranks ninth per million inhabitants [2]. The Brazilian Unified National Health System (*Sistema Único de Saúde—SUS*) pays for more than 95% of the transplants performed in the country [2], and it provides the necessary posttransplant medication and follow-up care, representing a growing demand upon public resources [3]. It may be the largest public transplant program worldwide [4].

Recipients of successful transplants have higher quality of life, which is directly linked to the continuous normal graft function [5]. Over the past two decades, significant progress has been achieved in graft rates and patient survival rates after kidney transplantation [6]. Some studies show that the half-life of deceased and living related allografts has improved to 13.8 and 21.6 years, respectively [7], and that there is more than 95% of patient survival rate and more than 91% of organ survival rate in one year [1].

Allograft rejection occurs because the recipient's immune system recognizes the donor's tissue as foreign and attacks the graft. Immunosuppressive agents may prevent or reverse most acute rejection episodes. However, even though these agents prevent acute rejection, the graft may still succumb to a more insidious type of chronic rejection characterized by replacement fibrosis of the graft parenchyma developing for over months or years [8].

As immunosuppressive agents have become more effective at controlling acute rejection, chronic rejection has emerged as one of the major problems in clinical practice. This process leads to an inexorable loss of kidneys at the rate of approximately 5% to 7% per year [9, 10].

Analysis of sequential renal transplant biopsies suggests that chronic rejection represents cumulative and incremental damage to the graft due to time-dependent immunologic and nonimmunologic causes, whereby chronic allograft dysfunction is the chief cause of kidney transplant failure [11, 12]. Most grafts are lost because of a continuous process characterized by interstitial fibrosis, tubular atrophy, and severe atherosclerosis [13, 14].

According to Banff classification, rejection can be mediated by both antibodies and T lymphocytes, and it may be acute or chronic [12]. C4d complement fragment was the antibody-mediated rejection marker [12]. Nevertheless, the low sensitivity of that marker was discussed in the last meeting in 2011 [15], as there may be an antibody-mediated rejection and leukocyte activation with antibody Fc receptors without activation of complement cascade, case in which there is no C4d deposition [15]. There might be changes in Banff classification concerning antibody-mediated rejection in the next meeting in 2013 in Brazil [15].

A chronic active T-cell-mediated rejection has arterial intimal fibrosis with mononuclear cell infiltration in fibrosis, formation of neo-intima, called chronic allograft arteriopathy [16]. In that case, lymphocytes seem to play a key role in the immune response of the graft, leading to tissue damages [17].

## 2. Immunology of Chronic Rejection

The immunological response involved in the chronic rejection process depends on both innate and adaptive immune response. Evidence has been drawing attention to the involvement of innate mechanisms, such as the complement system and the NK cells, in chronic rejection [18]. In adaptive immunity, antigen presenting cells (APCs) play an important role in directing the response of T lymphocytes, either by direct response (donor APC) or indirect response (recipient APC) [19]. It is assumed that the stimulation of T-cells through direct response tends to decrease over time as donor APCs are replaced by host APCs. Thus, the predominant immune response causing chronic rejection occurs through an indirect pathway. B lymphocytes have a major role in the development of chronic rejection, whether it may be acting as APCs, or as a source of cytokines, or by producing alloantibodies. The latter have a great potential of activation of the complement system and the binding mechanism at the Fc region in phagocytes [12]. On the other hand, regulatory B lymphocytes (also known as Bregs) may have an important part in promoting graft tolerance maintenance, both directly through regulatory cytokine production, such as IL-10, or by contributing to an increase in Tregs [20–25].

Studies in pigs suggested that vascular changes observed in chronic rejections are more likely to occur when disparities are observed in class I antigens rather than class II antigens, indicating that CD8+ T-cells are implicated in lesion development. In rat models, however, there is some pieces of evidence that both CD4+ and CD8+ cells are capable of causing lesions and that disparities in both class I or class II antigen presentation are crucial to induce chronic rejection [26]. Studies in mice have shown that class I disparities are enough to induce cardiac allograft pathology. That could be explained by the expression of MHC class II on the vascular endothelium of transplanted mice with vascular lesions [27]. Because MHC class II is not constitutively expressed by vascular endothelial cells of mice, indirect recognition of donor class II transferred from passenger leukocytes may be responsible for inducing an inflammatory response that leads to subsequent upregulation of class II on the vascular endothelium of the donor. Adoptive transfer studies in mice have revealed that in the absence of T-cells alloantibodies are able to induce vascular alterations that are typical of chronic rejection [28]. Nonetheless, another study showed that in the absence of B cells, T-cells do induce lesion development, but with less tendency to progress to a final stage of fibrosis [29]. Moreover, even in the absence of T-cells other cells such as NK cells could induce lesions in cardiac allografts [30]. Hence, it seems that various immunological mechanisms are capable of inducing characteristic lesions observed in chronic rejections, and that T-cells alone are not essential for their induction. So far, the existence of a single mediator that could be commonly involved in all these pathways is still unclear. Even though, cytokine IFN-*γ* seems to play a vital role in lesion development in many different chronic rejection models [27, 31]. Vasculopathy in STAT4-deficient mice, which are nonresponders to IL-12 stimulation and are incapable of generating Th1 responses, is less intense than the vasculopathy observed in wild-type mice [32]. On the other hand, the anti-inflammatory cytokine TGF-*β* would be important to attenuate lesion size, but because of its profibrotic role, TGF-*β* is highly expressed in vascular lesions caused by chronic rejections [33].

It is noteworthy that the definition of the immune response mechanisms involved in chronic rejections is still unclear, as the key molecules involved in the immunopathogenesis of this entity are still unknown. Meanwhile, we aim to present a general overview on the state-of-the-art knowledge of the strategies used for manipulating the lymphocyte activation mechanisms involved in allograft rejection, with emphasis on T lymphocyte costimulatory molecules. First, we will focus on the key molecules involved in the basic co-stimulatory and co-inhibitory processes of T-cell biology. Afterwards, we will discuss some of the most important experimental and clinical studies that shed some light on the increasing survival of solid transplanted organs, particularly kidney transplant.

## 3. T-Cell Immune Response: A Two-Signal Hypothesis and More

T lymphocytes are considered to be the key cells involved in host-cell immune response, mainly due to their ability to be activated in an antigen-specific manner and to potentiate components of both innate and adaptive immune responses. It has been demonstrated that only antigen-specific activation—mediated by TCR—was not enough to cause lymphocyte activation and, also, when it occurred alone it led to cell anergy and peripheral tolerance [34]. In addition, great importance has been given to the co-stimulatory and co-inhibitory signaling molecules which together with TCR signaling form a model initially called the *Two-signal hypothesis* [35]. Later studies showed that lymphocyte activation, besides being a process mediated by two signals (antigenic recognition and costimulation), depends on the coordinated interaction of various molecules called costimulators and coinhibitors, given the capacity such molecules have to mediate the stimulation or inhibition of specific antigen activation [36–38]. Nowadays it is known that costimulation of T helper cells is crucial for determining their phenotype. The different subtypes of effector T-cells are all generated from naïve T-cells according to the type and intensity of co-stimulatory signals recognized during the cell differentiation process. Furthermore, the action of effector cells on the periphery, although in a lesser extent, is still driven by the signals generated from the antigenic recognition by TCR. Taking these variable functions into account, it is of great interest to better understand the balance between T-cell costimulation and coinhibition events in chronic infections [39], tumors [40], autoimmune diseases [41], asthma [42], and allograft tolerance [43], as it might represent important therapeutic strategies.

## 4. B7-CD28 Superfamily

Although the interaction between B7-1 and B7-2 with CD28 or CTLA-4 is classically considered as the main co-stimulatory and co-inhibitory stimuli, many other molecules have been described to act in these processes. Because of their structural similarities these molecules are placed in large groups or families, such as B7-CD28 superfamily, TNF-TNFR superfamily, CD2 superfamily, Integrins superfamily, and TIM superfamily. This paper will focus on the key members of the B7-CD28 superfamily, their biology, and the promising intervention in their signaling pathways, which allows for the development of new therapeutic strategies capable of maintaining renal allograft survival for a long time after implantation in the host. The main members of the CD28 family and their principal roles are summarized in Table 1.

## 5. CD28 and CTLA-4

Traditionally, the relationship between T-lymphocyte costimulation and co-inhibition is demonstrated by CD28/CTLA-4 duality. Both molecules are expressed on the T-cell surface and share the same ligands, mainly B7-1 (CD80) and B7-2 (CD86), constitutively expressed on the cell surface of APCs and augmented on activated dendritic cells (DCs) [103]. In spite of their redundancy in the binding to B7-1 or B7-2, CD28 and CTLA-4 molecules signal antagonist functions in T lymphocytes [51, 104]: the interaction of B7-1/B7-2 with CD28 as well as antigen recognition via TCR both provide a continuous co-stimulatory signal to activate lymphocytes and induce the expression of antiapoptotic genes, for example, Bcl-2 and Bcl-XL [49, 50]. Consequently, this interaction also induces cell survival and proliferation and IL-2 production, and it potentiates the T-cell-dependent B-cell activation [51–54]. Nevertheless, the binding of the same molecules to CTLA-4 induces the expression of proapoptotic genes as well as the production of immunoregulatory cytokines, and it also favors restoration of homeostasis and induction of peripheral tolerance, both required for the prevention of autoimmune phenomena and for the survival of transplanted organs [40, 51, 53, 55–58].

 Although CD28 and CTLA-4 share the same ligands, their availability on the cell surface and their binding affinity to specific ligands may vary according to the differentiation stage of the cell or even according to the cell subtype: naïve T-cells express 10 times less CTLA-4 than CD28 on their surface [105]. After cell activation, the expression of CTLA-4 increases progressively [55, 106]. For example, CTLA-4 is constitutively expressed on the cell surface of regulatory T-cells, and its affinity to CD80 is increased [107, 108].

Studies have shown that the absence of CTLA-4 in regulatory T-cells leads to a noticeable loss of its suppressing functions, causing uncontrolled lymphoproliferative response, autoimmune disease, and increased production of immunoglobulins. This may be due to the fact that CTLA-4-expressing Tregs reduce CD80 and CD86 expression in antigen-presenting cells [78, 109]. Mice lacking CD28 or CD80/CD86 expression are extremely susceptible to infections [110, 111], while CTLA-4-deficient animals develop severe immunopathologies a few weeks after birth [112–114]. This effect might be prevented by administration of CTLA-4-Ig [115].

## 6. ICOS

Another co-stimulatory molecule that belongs to the CD28-superfamily is ICOS [59], whose unique ligand is called ICOSL [116–118]. The ICOS molecule is a coreceptor which is expressed at low levels on naïve lymphocytes, but which is upregulated after TCR and CD28 stimulation [44]. Moreover, ICOS expression has been correlated with the T-cell differentiation into Th1, Th2, Th17, Tfh, and Treg lymphocytes [44–46], but with distinct upregulation mechanisms for each subpopulation [45, 119]. Among the main effects of ICOS-ICOSL binding is the induction of T-cell proliferation with increased IL-2, IL-4, IL-5, IL-10, IFN-*γ*, TNF-*α*, and GM-CSF production [44, 59, 60]. Some studies performed *in vivo* showed that the absence of ICOS makes the animals more susceptible to viral and helminthic infections, mainly due to a considerable decrease in Th1 and Th2 responses [120]. The lack of ICOS expression also inhibits the development of autoimmune diseases and airway inflammation [121, 122], but it induces an important loss of memory T-cell function [123]. Interestingly, ICOS expression seems to be crucial for the development and homeostasis of Tregs [61, 62], while deficient mice had some defects in tolerance induction [124].

## 7. PD-1

Like CTLA-4, PD-1 molecule is a member of the CD28 superfamily whose functions are related to the inhibition of immune responses [63, 64]. Unlike other family members, PD-1 is expressed over many developmental stages of CD4+ and CD8+ T lymphocytes, B lymphocytes, monocytes, and NK cells [47, 48]. It binds to at least two ligands denominated PD-L1 and PD-L2, which are expressed on a large variety of cell types, such as DCs, T and B lymphocytes, mesenchymal stem cells, mast-cells, and other nonhematopoietic cells [125, 126]. PD-1 is a potent inhibitor of cell proliferation and cytokine production [65–67], being capable of inhibiting T-cell activation even at low concentrations [127]. In addition, PD-1 participates in the suppressive processes mediated by Tregs [71] so it is an important molecule in maintaining peripheral cell tolerance, which is essential to avoid autoimmune reactions and to allow the graft to increase its survival rate [68–70]. Interestingly, other studies have shown that PD-L1 and PD-L2 may contribute to the co-stimulatory signals of T lymphocytes, raising the question of whether these molecules have ligands other than PD-1 [128–130].

Many other molecules were and still have been found to play an important role in the co-stimulatory or co-inhibitory processes that rule T-cell biology. The next section of this paper is devoted to the role of these molecules both in the host response to the allografts and in potential interference strategies in allogeneic transplant models.

## 8. Role of a Second-Signal on Allogeneic Immune Response: *In Vitro* and *In Vivo* Approaches

In a recent revision by Vicentini (2008), since the 1970s the description of the *two-signal hypothesis* was crucial for lymphocyte development and the effector function [35], as many models have been used to evaluate the diverse strategies used in the manipulation of the co-stimulatory or co-inhibitory molecules in animal models. These strategies are crucial for understanding the role played by these signals in response to allografts, as well as for the development of new drugs aiming to increase graft survival or to reduce the deleterious effects of autoimmune reactions, especially by interfering with B7-1/B7-2 and CD28 pathways [86, 131–137].

## 9. From Mice to Belatacept

As aforementioned, the interaction of CD28 with B7-1 and B7-2 molecules is the usual co-stimulatory signal, while the interaction of CTLA-4 with its receptors provides a stop signal for T-cell activation stages. Since initial studies, the interference on these pathways has been deemed a useful strategy to fight autoimmune reactions and graft rejection or to upregulate the immune response against chronic infections. Nevertheless, it is noteworthy that an interference in the CD28 signaling pathway alone seems to be unable to prove what preliminary studies suggested: that CD28^−/−^ animals are still capable of rejecting a graft [72], yet after a longer time than expected for a wild-type animal, which suggests that other co-stimulatory pathways might be important for graft rejection [72, 73]. It is also clear that adult animals whose CD28 co-stimulatory pathway is blocked obtain better results regarding graft tolerance and survival rates [81], emphasizing that knockout animals probably develop other compensation strategies in the absence of CD28 signals. On the other hand, CTLA-4 expression in experimental models seemed to be more related to the number and function of allospecific T-cells and to the increasing number of Tregs [78, 79]. The administration of anti-CD45RB antibodies, which increases the number of CTLA-4 molecules, leads to a temporary increase in CTLA-4-dependent tolerance to allografts [80].

Essentially, three approaches have been used to interfere with CD28 signaling pathway and to increase allograft survival: use of anti-CD28 or anti-B7-1/B7-2 blocking antibodies, the administration of CTLA-4-Ig, a fusion protein of CTLA-4, and Fc of immunoglobulin (Ig).

Experimental models using anti-CD28 antibodies showed the longest tolerance time to kidney transplants [74], reduced donor cell reaction, and a synergistic effect between these antibodies and rapamycin in GVHD models [75]. Interestingly, CD28 stimulatory antibodies also demonstrated to be potential inhibitors of the host response against allografts, mainly due to an increase in the apoptosis rate of effector cells [75–77]. Haspot and colleagues observed a better response and an increased tolerance to kidney transplants concerning IDO and iNOS (inducible nitric oxide synthase) [138]. Conversely, a clinical trial adopting this strategy showed that the administration of anti-CD28 in six volunteers initiated a systemic inflammatory response [139], probably due to a huge secretion of proinflammatory cytokines by stimulated T-cells [140]. It is noteworthy to mention that this syndrome has never been found in experimental models involving nonhuman primates [75–77].

The administration of anti-B7-1/B7-2 antibodies showed it is capable to decrease the rejection rate of kidney transplants in animal models [141, 142] and in preclinical human studies [143], but despite these good initial results, this approach has not evolved into clinical trials [144].

Nowadays, the usual strategy is to block the co-stimulatory pathway through the administration of CTLA-4-Ig, a fusion protein consisted of the Fc region of immunoglobulin G and of the extracellular domain of the molecule CTLA-4, initially named abatacept [133, 145], that acts as a competitor in the binding of CD28 to B7-1/B7-2 [108].

Initial studies demonstrated that *in vitro* addition of these molecules completely blocked the proliferation of T-cells against alloantigens [81], induced cell anergy [82], and decreased the T-cell-dependent antibody response [145]. Studies on experimental models of cardiac transplantation showed promising results on the use of CTLA-4-Ig by blocking or even postponing the rejection process [83–85]. In rodent models of transplantation, the administration of CTLA-4-Ig or anti-CD80 and anti-CD86 antibodies for a short period of time was able to prevent the rejection of MHC-mismatched heart, kidney, and pancreas [133, 146–149], although the same results could not be observed in skin transplants or in nonhuman primate models, when only a slight increase in graft tolerance was observed [150–152]. A reasonable explanation for such modest results would be that avidity of abatacept to B7-2 is weaker than to B7-1 [137], and, thus, does not block CD28-B7-2 interaction, previously described as vital in the initiation of alloimmune reaction [153]. This binding difference was crucial to the need of a second generation of CTLA-4-Ig to be developed, which was named LEA29Y or belatacept. This new molecule was obtained from a mutation in two positions (L104E and A29Y), which resulted in a ten times stronger affinity of belatacept to B7-1 and B7-2 binding than that obtained by abatacept [137].

Although it had been indicated that B7-2 blockade would allow for a more effective induction of tolerance to grafts than the blockade of B7-1 [154], when both molecules were simultaneously blocked, however, better results were achieved [153].

Some recent studies have shown that belatacept has other actions rather than decreasing the number of effector T-cells or increasing the function of Treg cells [155, 156]. However, *in vitro* studies showed that belatacept can lower the number of naïve and effector T-cells and that the modified Fc region of the molecule is unable to induce either complement-dependent or antibody-dependent cytotoxicity phenomena [157].

By evaluating the potential influence of proinflammatory cytokines on belatacept action, Zhao and colleagues suggested that IL-6 participates in allograft response and that the modulation of this cytokine production may increase tolerogenic potential of belatacept administration [158]. Previously, it has been demonstrated that CTLA-4-Ig regulates the expression of IDO—Indoleamine 2,3-dioxygenase—an enzyme that degrades tryptophan and deactivates T lymphocytes [159]. A recent study in humans showed an increase in the number of Tregs and CD16+IDO+ cells in belatacept-treated patients when compared with other patients who received cyclosporine treatment [87]. In experimental models, it could be noticed that the effect of belatacept depends on a combined action of IDO and Tregs [160].

Clinical studies comparing the efficacy of various belatacept regimens and cyclosporine treatment—in both cases the drugs were associated with *basiliximab induction therapy *(IL2R blocker)*, mycophenolate mofetil,* and* steroids*—showed that after six months of transplant the acute rejection rate was similar regardless of the chosen drug. Nonetheless, the group treated with a more intense regimen of belatacept had better renal function and reduced chronic allograft nephropathy rate [86, 88, 89]. Furthermore, side effects of belatacept in comparison with those of cyclosporine or of other calcineurin inhibitors are less intense with a reduced number of events such as leukopenia, anemia, edema, urinary tract infection, hypokalemia, acidosis, diabetes mellitus, and hypertension [86]. Another study evaluating 1,425 patients also observed better results with belatacept over cyclosporine administration [161]. However, Rostaing and colleagues could not observe huge differences among groups of patients treated with cyclosporine, belatacept, or tacrolimus [162].

The combination of rATG, belatacept, and sirolimus showed better *in vitro* and *in vivo* results, with higher survival rate of Treg cells and greater inhibition of alloreactive effector T-cell response after one year of treatment [90]. Another recent clinical study showed that patients who received belatacept alone, without an immunosuppressive drug or short-course tacrolimus immunosuppression, had better renal function and lower acute rejection rates in comparison with the patients who received tacrolimus for a longer period of time [163].

It is remarkable that notable advances have been achieved by recent clinical trials using the aforementioned strategies. Nevertheless, more attention should be drawn to the real benefits and the potential risks the wide administration of these drug regimens lead to, especially after the occurrence of posttransplant lymphoproliferative disorders (PTLD) in EBV-positive patients treated with belatacept [164, 165].

## 10. The Other CD28 Family Members

**ICOS-ICOSL Pathway**

Despite the clear understanding of its exact role in transplantation, a recent study has suggested that the ICOS-ICOSL co-stimulatory pathway might be relevant in the allograft rejection process [91]. Many murine models suggested that an increased graft survival rate would be observed after administration of blocking antibodies directed to ICOS or the administration of ICOS-Ig in hepatic [92] and cardiac transplants [93] and in GVHD [94, 95]. These strategies also prevented the development of chronic allograft vasculopathy [96], but they were unable to prevent graft rejection in a model of kidney transplant [98]. This discrepancy in the results seems to be related to the timing of ICOS blockade and to the concomitant blockade of the CD28 pathway. In addition, studies in mice suggested that early administration of anti-ICOS has more modest results in prolonging graft survival than late administration, when the suppressive effects of CD8+ T-cells were more pronounced [61]. Rodent experimental models of cardiac allografts had an increased graft survival rate when ICOS blockade was associated with cyclosporine, CTLA-4-Ig, or anti-CD40L (CD40 receptor, a co-stimulatory member of the TNF-TNFR family) [64, 96, 97]. Functionally, ICOS blockade suppresses the activation and the cytokine production of alloreactive T-cells [96], and it interferes with memory T-cell recruitment to the allograft [166]. Interestingly, Hodgson and colleagues observed that the administration of anti-ICOS provides a better tolerance to xenotransplant, characterized by an increasing number of infiltrating Tregs in the transplanted tissue [167]. More importantly, though, is the wide distribution of ICOS molecules within the T-cell subpopulations, having many key roles in the development of effector and regulatory immune responses [44–46]. Such observation may limit the clinical use of ICOS-ICOSL inhibitors once the blockade of this signaling pathway would deplete important mechanisms involved in graft tolerance.

**PD-1-PD-L1/PD-L2 Pathway**

The interaction between PD-1 and its ligands seems to play a pivotal role in the regulation of alloimmune responses in animal models [99–101], when the blockade of this interaction leads to rapid allograft rejection [168]. The administration of anti-PD-1 blocking antibodies reduces the deleterious effects of GVHD [169]. Interestingly, however, are the conflicting results obtained by the administration of blocking antibodies directed to other molecules of the PD-1 pathway. While anti-PD-1 treatment increases graft survival rate, the administration of anti-PD-L1 antibodies accelerates the rejection process [101]. These results could be explained by the fact that PD-1 interaction with PD-L1 rather than with PD-L2 is vital for maintaining graft tolerance [170, 171]. Furthermore, the effects observed by the blockade of PD-1 interaction with its ligands seem to depend on the CD28 co-stimulatory pathway [172]. Additionally, it has been shown that besides PD-1-PD-L1 interaction, the binding of PD-1 to B7-1 also plays an important role in the inhibition of alloimmune responses [173]. Other studies have pointed out an important role of PD-1 ligands, especially PD-L1, in graft survival in many transplant models. More recently, PD-L1 expression in transplanted tissues has been implicated in the induction of graft-host tolerance [102], probably by controlling the deleterious effects of ischemia and reperfusion injury [174, 175] or by suppressing alloreactive T-cells in kidney transplants [176]. Apart from the aspects regarding rejection physiopathology, new studies correlate PD-1 expression or some functional polymorphisms of this molecule with an increased risk of cytomegalovirus infection in patients undergoing kidney or liver transplantation [177–181]. Likewise, the determination of mRNA expression in biological fluids of PD-1 as well as in other co-stimulatory molecules appears to be a further strategy capable of predicting acute renal rejection [182–186].

## 11. Immunological Barriers to Effective Costimulation Blockade

Although we have been noticing promising results regarding blockade strategies of co-stimulator molecules as a therapy for graft tolerance maintenance in the past years, such strategy does not always have positive outcomes. A few individuals are resistant to costimulation blockade-induced tolerance. Some intrinsic factors of the receptor or factors related to cells from the donor may hinder the success of this therapy. Two important situations may have a negative impact on the success of costimulation blockade: the donor-reactive T-cell precursor frequency and the resistance of receptor memory T-cells to the mechanisms of costimulation blockade.

It has been demonstrated for some time that different T-lymphocyte subclasses have distinct sensitivities to costimulation blockade as a tolerance inducer, particularly CD8 T-cells + graft [187, 188]. Moreover, it has been demonstrated that the frequency of donor-reactive T-cells may hinder the interference of costimulation blockade in the generation of effector T-cells, and that such generated cells may lead to graft rejection [144]. Important studies by Ford and colleagues showed that, at a low frequency, donor-reactive T-cell precursors are susceptible to costimulation blockade, interfering with the generation of graft rejection effector cells. On the other hand, a high donor-reactive T-cell precursor frequency is resistant to the action of costimulation blockers, which may generate effector cells even in the presence of blockers [189–191].

Another relevant possibility that may hinder the success of the therapy with co-stimulator blockers is the previous occurrence of donor MHC-peptide complexes in the receptor memory T-cells with cross-reactivity. These cells can be generated by prior transplantation, blood transfusion, pregnancy, or environmental exposure to pathogens [144, 192]. In fact, many studies have shown that the concomitant occurrence of some types of infections leads to decreased graft tolerance [192–197]. Typically, memory cells have less need of activation signals than naïve cells, both signals arising from antigen and from co-stimulator molecules [198, 199]; hence, they are also resistant to the effects of costimulation blockade. The right understanding of the role of donor-reactive T-cell precursors and of memory cells with cross-reactivity to donor MHC-peptide complexes in the rejection process, as well as the right understanding of how such obstacle might be overcome, can improve the success of costimulation blockade strategies to a greater extent.

## 12. Concluding Remarks

As described above, co-stimulatory molecules play a pivotal role in immune response control. Over the years, many molecules and surface receptors have been described as potent controllers of both effector or regulatory T-lymphocyte development and function. A deeper understanding of the structure and function of these molecules improves both the knowledge of the immune system itself and also their potential action as rejection inducers or tolerance promoters. In this context, the central role played by CD28 family, especially the relationship between CD28 and CTLA-4, becomes an interesting target to the development of immune-based therapies aiming to increase allograft survival rate and to decrease autoimmune phenomena. Good results obtained by the recent development of drugs with potential clinical use aroused better expectations concerning the outcome of transplanted patients, even though large-scale studies are still needed to confirm their safety and advantage over traditional therapies. Furthermore, the manipulation of the signaling pathway of other members of the CD28 family, such as ICOS and PD-1, may provide new perspectives for additional therapies and also for important markers of graft survival.

## Figures and Tables

**Table 1:** Expression of CD28 family members, ligands, and targeting in transplantation.

Receptor	CD28	CTLA-4	ICOS	PD-1
Expression	Constitutive on T-cells	Induced on T-cells	Induced on Tcells after TCR and CD28 stimulation [44]. Th1, Th2, Th17, Tfh, and Treg lymphocytes [44–46]	T lymphocytes, B lymphocytes, monocytes and NK cells [47, 48]
				
Ligand	CD80 (B7.1); CD81 (B7.2)	CD80 (B7.1); CD81 (B7.2)	ICOS-L (CD275, B7-H2, B7h, B7RP-1)	PD-L1 (CD274, B7-H1); PD-L2 (CD273, B7-DC)
				
Ligand expression	APCs, activated dendritic cells (DC) B cells, T-cells, monocytes	APCs, activated dendritic cells (DC) B cells, T-cells, monocytes Nonlymphoid cells (liver, ling, kidney)	APCs, activated dendritic cells (DC) B cells, T-cells, monocytes Nonlymphoid cells (liver, lung, kidney)	DCs, T and B lymphocytes, mesenchymal stem cells, mast-cells and other nonhematopoietic cells [125, 126]
				
Major role	Positive costimulation expression of antiapoptotic genes [49, 50] cell survival, proliferation, IL-2 production and potentiates the T-cell-dependent activation of B lymphocytes [51–54]	Negative costimulation Expression of the proapoptotic genes Production of immunoregulatory cytokines Peripheral tolerance [40, 51, 53, 55–58]	Positive costimulation induction of T-cell proliferation, augmented cytokine production [44, 59, 60] Generation and homeostasis of Treg cells [61, 62]	Negative costimulation [63, 64]. Inhibitor of cell proliferation and cytokine production [65–67]. Peripheral cell tolerance [68–70]. Suppression mediated by Treg [71]
				
Role in Allograft Rejection	CD28^−/−^-delayed graft rejection [72, 73] CD28 co-stimulatory blockade—graft survival [74–77]	↑ number of Treg cells [78, 79]. Augmented number of CTLA-4 molecules—↑ tolerance to allografts [80] CTLA-4-Ig—↓ proliferation of allospecific T-cells [81, 82]; prevent the rejection [83–85]; ↑ graft survival in human transplantation [86–90]	Allograft rejection [91] ICOS-Ig—↑ graft survival [64, 92–97]; ↓ kidney transplant survival [98]	Regulation of alloimmune responses in animal models [99–101]. Anti-PD-1 treatment—↑ graft survival anti-PD-L1 antibodies ↑ rejection process [101]. PD-L1 expression in transplanted tissues—tolerance to the graft [102]
